# Effect of Ozone Autohemotherapy on Inflammatory Response and Postoperative Cognitive Function in Patients Undergoing Valve Replacement with Cardiopulmonary Bypass

**DOI:** 10.21470/1678-9741-2024-0313

**Published:** 2025-09-11

**Authors:** Wenyong Peng, Yulong Du, Wei Lu, Xiaofeng Jiang, Hua Chai, Pengsheng Tian, Danyan Zhu

**Affiliations:** 1 Department of Anesthesiology, Affiliated Jinhua Hospital, Zhejiang University School of Medicine, Jinhua, Zhejiang, People’s Republic of China; 2 School of Medicine, Shaoxing University, Shaoxing, Zhejiang, People’s Republic of China; 3 Department of Prevention and Health Care, Affiliated Jinhua Hospital, Zhejiang University School of Medicine, Jinhua, Zhejiang, People’s Republic of China; 4 Department of Anesthesiology, Fuwai Hospital, Chinese Academy of Medical Sciences, National Center for Cardiovascular Disease, Beijing, People’s Republic of China

**Keywords:** Oxygen Saturation, Cardiopulmonary Bypass, Cardiac Valve Replacement, Inflammatory Response, Postoperative, Cognitive Dysfunction.

## Abstract

**Objective:**

We herein probed the effects of ozone autohemotherapy (O3-AHT) on inflammatory response and postoperative cognitive function in patients undergoing valve replacement with cardiopulmonary bypass (CPB).

**Methods:**

Totally, 130 patients undergoing valve replacement with CPB were included in the study (O3-AHT) and control (banked blood transfusion) groups. Blood samples were taken for blood gas analysis, with arterial oxygen saturation, jugular venous oxygen saturation, partial pressure of arterial oxygen and jugular venous PO₂, hemoglobin, and cerebral oxygen extraction rate documented. Interleukin (IL)-6, tumor necrosis factor alpha (TNF-α), and IL-1β levels and serum S100β and neuron-specific enolase (NSE) concentrations were measured by enzyme-linked immunosorbent assay, followed by cognitive function assessment by Mini-Mental State Examination and Montreal Cognitive Assessment scales.

**Results:**

The research group exhibited elevated thrombin time, activated partial thromboplastin time, and prothrombin time and decreased fibrinogen level immediately after surgery; it also presented reduced 24-hour postoperative serum IL-6, TNF-α, IL-1β, S100β, and NSE levels. Intraoperative cerebral oxygen metabolism was improved, and cognitive dysfunction was alleviated in the research group. The comparison of transfusion complication incidence between the two groups showed no significant difference.

**Conclusion:**

The application of O3-AHT in patients undergoing valve replacement with CPB enhanced intraoperative brain oxygen metabolism and reduced postoperative 24-hour inflammatory response and cognitive dysfunction.

## INTRODUCTION

**Table:** 

Abbreviations, Acronyms & Symbols				
APP	= Amyloid-β precursor protein		MB	= Myoglobin
APTT	= Activated partial thromboplastin time		MMSE	= Mini-Mental State Examination
BMI	= Body mass index		MoCA	= Montreal Cognitive Assessment
CaO_2_	= Arterial blood oxygen content		NSE	= Neuron-specific enolase
CERO_2_	= Cerebral oxygen extraction rate		NT-proBNP	= N-terminal B-type natriuretic peptide
CjvO_2_	= Jugular venous oxygen content		O3-AHT	= Ozone autohemotherapy
CK-MB	= Creatine kinase-muscle and brain isoenzyme		PaO₂	= Partial pressure of arterial oxygen
CPB	= Cardiopulmonary bypass		P_et_CO₂	= End-tidal carbon dioxide partial pressure
CVP	= Central venous pressure		PjvO₂	= Jugular venous oxygen pressure
ELISA	= Enzyme-linked immunosorbent assay		PT	= Prothrombin time
FIB	= Fibrinogen		rSO₂	= Regional cerebral oxygen saturation
Hb	= Hemoglobin		SaO₂	= Arterial oxygen saturation
Hs-cTnT	= High-sensitivity cardiac troponin T		SjvO₂	= Jugular venous oxygen saturation
IL	= Interleukin		SpO₂	= Oxygen saturation
LAD	= Left atrial diameter		TNF-α	= Tumor necrosis factor alpha
LVEDD	= Left ventricular end-diastolic dimension		TT	= Thrombin time
LVEF	= Left ventricular ejection fraction			

Cardiac valve replacement, a frequently performed procedure in cardiac surgery, encompasses various types such as mitral valve replacement, double valve replacement, and aortic valve replacement^[1,2]^. Cardiopulmonary bypass (CPB) is a commonly conducted procedure in cardiovascular surgery and is crucial for managing cardiac valve disease, coronary heart disease, and congenital heart disease^[3]^. CPB in cardiac surgical procedures allows for significant surgical advancements by providing the opportunity to operate on a quiescent heart in a less blood condition, while maintaining systemic perfusion and oxygenation^[4]^. Nevertheless, CPB can induce coagulation disorders and systemic inflammatory response, potentially resulting in adverse clinical outcomes^[5,6]^.

Ozone autohemotherapy (O3-AHT) is a medical procedure that involves combining autologous blood with a suitable quantity of anticoagulant and ozone before reintroducing it into the body, and this process leverages the pharmacological properties of ozone to stimulate red blood cell metabolism, enhance the immune system, and boost the antioxidant system^[7]^. Recent research has indicated that ozone can alter the process of platelet aggregation in the blood, and in the context of thrombosis, ozone plays a part in shifting the aggregation towards disintegration by producing hydrogen peroxide, consequently impacting the individual’s coagulation function^[8,9]^. Moreover, ozone activates superoxide dismutase, enhances *in vivo* free radical scavenging, and enhances platelet polymerization, thrombus dissolution, and blood vessel smoothness^[10]^. For instance, O3-AHT activates the pentose phosphate pathway to increase the 2,3-diphosphoglycerate content in erythrocytes, shift the oxygen dissociation curve to the right, and increase the oxygen supply to peripheral tissues. The peroxidative property of ozone augments the oxygen-carrying capacity of erythrocytes in the bloodstream, mitigates the excessive production of free radicals, and enhances blood flow to vital organs^[11-14]^. O3-AHT not only effectively enhances blood rheology but also avoids immune transfusion reactions and cross-infections caused by allogeneic blood transfusion and is widely utilized in the treatment of insomnia, neuropathic pain, gout, pneumonia, and other diseases^[15-19]^.

Although with advancements in modern medicine, the adverse effects of cardiac surgical procedures have been notably improved, concerns remain extant for the risks associated with CPB undertakings, including intraoperative issues and cognitive impairment^[20,21]^. Currently, cerebral oxygen extraction rate (CERO_2_) is utilized for evaluating the correspondence between cerebral blood flow and cerebral oxygen consumption^[22,23]^. Regional cerebral oxygen saturation (rSO_2_) can serve as an indicator of supply-demand balance, as well as changes in cerebral blood flow, and it is less susceptible to the influence of low temperature factors and non-ambulatory blood flow, making it a suitable method for monitoring cerebral oxygenation during cardiopulmonary circulation^[24,25]^. Importantly, postoperative cognitive dysfunction may be anticipated through intraoperative monitoring of the extent of decline in rSO_2_, a practice advocated as a standard assessment for elderly patients undergoing cardiac surgery^[20,26]^. Additionally, the reductions of perioperative inflammatory response and postoperative cognitive dysfunction in patients continue to be a significant clinical concern. Low concentrations of ozone have the potential to activate the immune system, while medium and high concentrations contribute to immunosuppressive effects, and ozone’s dual regulatory capacity is beneficial for restoring the body’s immune equilibrium, and all of the changes can positively influence the inflammatory response and postoperative cognitive function in patients undergoing valve replacement with CPB^[27,28]^. It has been documented that O3-AHT mitigates secondary myocardial injury during traumatic brain injury, which may be linked to its myocardial protective property against oxidative stress^[29]^. Moreover, ozone treatment has been shown to prevent in-stent coronary artery intimal hyperplasia, which may be attributed to the stimulation of the redoxin system by ozone pretreatment, which appears to neutralize oxidative damage from the outset and enhance antioxidative buffering capacity following injury, mitigating further damage and reducing the demand for antioxidant enzymes^[30]^. Therefore, O3-AHT has potential advantages in the management of patients undergoing valve replacement with CPB. Currently, the prevailing literature predominantly concentrates on the risk factors associated with delirium following valve replacement with CPB, as well as the management of CPB and surgical methodologies. However, the application of O3-AHT in cardiac surgery has not been extensively researched, and its potential advantages within this field are not fully defined. Therefore, this study aimed to evaluate the clinical efficacy of O3-AHT on patients undergoing valve replacement with CPB, especially the effects on their postoperative inflammatory response, coagulation function, cerebral oxygen metabolism, and cognitive function, offering new ideas for blood transfusion operations in patients undergoing cardiac surgery under CPB.

## METHODS

### Ethics Statement

The patients and their families were fully informed and signed the informed consent preoperatively. The study was reviewed and approved by the Medical Ethics Committee of Affiliated Jinhua Hospital, Zhejiang University School of Medicine (Research 2021- Ethical Review -172) in accordance with the Declaration of Helsinki.

### Study Subjects

The sample size for this study was estimated using G*Power 3.0.10 software (University of Düsseldorf, Nordrhein-Westfalen, Germany) based on a statistically efficient approach. First, the statistical parameters were set as a two-tailed test (α = 0.05, 1-β = 0.8), with a predicted medium effect size (effect size d = 0.5) and a sample size ratio of n1/n2 = 1 (n1 represented the control group, n2 represented the research group). The estimated results were n1 = 64 and n2 = 64, and the total sample size was n = 128. Considering an anticipated loss rate of approximately 20%, the initial sample size for inclusion was set at 212 cases. Accordingly, a total of 212 patients who underwent cardiac valve replacement with CPB from June 2020 to June 2023 by the same group of surgeons at the Department of Cardiac Surgery of Affiliated Jinhua Hospital, Zhejiang University School of Medicine were selected. Among these, 45 cases did not meet the inclusion criteria, 27 cases declined to participate in the study, three cases had incomplete information, and seven cases withdrew from the study. Finally, 130 patients were included as the study subjects and then randomly divided into the research group (n = 65) and the control group (n = 65). O3-AHT was applied in the research group, banked blood transfusion was utilized in the control group, and other blood protection measures were the same in both groups.

### Inclusion and Exclusion Criteria

Inclusion criteria were as follows: 1) preoperative New York Heart Association Functional Classification grade II to III and American Society of Anesthesiologists grade II to III; 2) the need for valve replacement with CPB; 3) first time undergoing cardiac surgery; 4) 18 to 70 years old; 5) no history of symptomatic treatment within 14 days prior to enrollment.

Exclusion criteria were: 1) anemia, blood disorders, or abnormal coagulation function; 2) preoperative major systemic diseases or systemic infections; 3) complication of thyroid dysfunction or immune deficiency; 4) use of anticoagulant and antiplatelet drugs within the last month; 5) recent use of glucocorticosteroids or other medications affecting inflammatory response; 6) a history of cardiac surgery within one year and surgery within three months prior to enrollment; 7) allergic to ozone or glucose-6-phosphate dehydrogenase deficiency; 8) significant cognitive dysfunction or inability to cooperate in completing the cognitive function assessment scale due to disability or other physical factors prior to the surgery; 9) left ventricular ejection fraction (LVEF) < 40% or symptoms of heart failure; 10) died in 24 hours postoperatively.

### Data Collection and Indicator Monitoring

The patients’ sex, age, body mass index, education background, comorbidity, and preoperative heart functional classification were acquired, and the patients’ CPB time, aortic occlusion time, along with intraoperative vital signs, including mean arterial pressure, heart rate, nasopharyngeal temperature, central venous pressure (CVP), oxygen saturation (SpO_2_), end-tidal carbon dioxide partial pressure (P_et_CO_2_), and rSO_2_, were recorded. The documented time points were as following: pre-anesthesia induction (T_1_), 10 minutes after anesthesia induction (T_2_), after ascending aorta occlusion (T_3_), 10 minutes after cooling to 30°C and stabilizing (T_4_), immediately after rewarming to 37°C (T_5_), at the end of CPB (T_6_), and immediately after operation (T7)^[31,32]^. At the same time, blood samples were collected via the radial artery and jugular bulb for blood gas analysis. Arterial oxygen saturation (SaO_2_) and jugular venous oxygen saturation (SjvO_2_), partial pressure of arterial oxygen (PaO_2_), jugular venous oxygen pressure (PjvO_2_), and hemoglobin (Hb) were recorded.


$$\begin{array}{rl} & {\mathrm{CERO}}_{2}=\left({\mathrm{CaO}}_{2}-{\mathrm{CjvO}}_{2}\right)/{\mathrm{CaO}}_{2}\text{, where }{\mathrm{CaO}}_{2}=(\mathrm{Hb}\times 1.36\times {\mathrm{SaO}}_{2}+\\ & 0.0031\times {\mathrm{PaO}}_{2})\text{, and }{\mathrm{CjvO}}_{2}=\left(\mathrm{Hb}\times 1.36\times {\mathrm{SjvO}}_{2}+0.0031\times {\mathrm{PjvO}}_{2}\right). \end{array}$$

where CaO_2_ is arterial blood oxygen content and CjvO_2_ is jugular venous oxygen content

### Conditions for Intraoperative Red Blood Cell Transfusion

(1) Patients with Hb < 80 g/L before or after CPB should receive red blood cell transfusion; (2) during CPB, patients with Hb < 60 g/L or patients with cerebral ischemic risk (history of cerebrovascular disease, diabetes, cerebrovascular disease, carotid artery stenosis) and Hb < 60 g/L should receive red blood cell transfusion; patients with Hb < 70 g/L but estimated to rise to Hb > 80g/L after ultrafiltration was turned off should not receive red blood cell transfusion; (3) Hb of patients with following conditions should be increased reasonably: limited heart and lung function and active bleeding or ischemia of vital organs indicated by laboratory or clinical indexes (mixed venous SpO_2_, electrocardiogram, or echocardiography); (4) patients with Hb > 100 g/L should not receive red blood cell transfusion unless for new ischemia of vital organs.

### Methods of Blood Transfusion

Before surgery, patients in both groups received nutritional support with ferrous succinate tablets (batch no.: 180502, Hunan Warrant Pharmaceutical Co., Ltd., Changsha, China) or ferrous lactate syrup (batch no.:1804030203, Shijiazhuang Yuhui Pharmaceutical Co., Ltd., Shijiazhuang, China). Patients in both groups were subjected to general anesthesia with tracheal intubation using 2 µg/kg fentanyl (batch no.: 1170806, Yichang Humanwell Pharmaceutical Co., Ltd., Yichang, China) and 0.2 mg/kg cisatracurium besylate (batch no.: 18012821, Jiangsu Hengrui Pharmaceutical Co., Ltd., Liangyugang, China) and maintained in anesthesia state using propofol (batch no.: 1707049, Beijing Fresenius Kabi Pharmaceutical Co., Ltd., Beijing, China), 1% sevoflurane (batch no.: 18012831, Jiangsu Hengrui Pharmaceutical Co., Ltd.) and 0.02-0.10 µg/ (kg-min) fentanyl (batch no.: 1171219, Yichang Humanwell Pharmaceutical Co., Ltd.). Surgery of patients in both groups was performed by the same group of surgeons after general anesthesia.

The O3-AHT technique was utilized in the research group, with electrocardiogram, blood pressure, and SpO_2_ of pulse detected routinely, and indicators such as mean arterial pressure, CVP, and electrolytes continuously monitored intraoperatively. Autologous blood salvaging device (Model BW-8200A, Wandong Health Sources, Beijing, China or Model 3000P, Beijing Jingjing Medical Equipment Co., Ltd., Beijing, China) was used to suck the blood from surgery wound and CPB tubes using negative pressure. Heparin (200 mg) was added into 500 mL normal saline to prevent coagulation. The coagulation filter, centrifuge chamber, blood collection bags, and waste bag were installed in the device and the blood storage reservoir was washed. The filter and double-lumen suction tubes were washed preoperatively with 0.9% normal saline (500 mL) and heparin (25000 U). The blood lost from the time of skin incision was salvaged using negative pressure system of double-lumen suction tubes to suck the blood into the blood storage reservoir. Once the blood in the reservoir reaches 800 mL, the device will automatically lead the blood to the centrifuge chamber at the speed of 600 mL/min for centrifugation at 10000 r/min, which will wash away the plasma, free Hb and anticoagulants. The red cell suspension was placed in blood collection bags and mixed with 25 µg/mL ozone (O3) of equal volume produced by an ozone generator (Model Medozon Comfort, Herrmann Apparatebau GmbH) for one to two minutes before the blood was reinfused into the patient. About one hour later, patients receiving reinfusion should receive routine blood tests. The drip rate of heparin/normal saline needs to be adjusted according to the individual bleeding volume to make sure the volume of heparin/normal saline with reinfusion blood was maintained at 1:100. In case of drainage volume exceeding 600 mL, multiple reinfusion is applicable (no more than 2000 mL of reinfusion per patient). The preoperative preparation and intraoperative observation of the control group were the same as those of the research group. The allogeneic blood of each patient in the control group was prepared according to their blood type before operation and transfused by intravenous drips based on bleeding volume during the operation.

### Regional Cerebral Oxygen Saturation Detection

rSO_2_ value of all patients was measured using a rSO_2_ monitor (Model MC-2030C, CAS Medical Systems Inc., United States of America). After the patients were admitted to the room, the forehead skin of patients was repeatedly wiped with alcohol for adequate degreasing. After placing the left and right sensors below the prefrontal hairline bilaterally, the patients were secured with adhesive tape to dynamically monitor rSO_2_. rSO_2_ values at T_1_ were set as the baseline value by recording the mean value of the left brain and the right brain.

### Enzyme-Linked Immunosorbent Assay

The fasting elbow venous blood (5 mL) 24 hours preoperatively and postoperatively were obtained in the early morning and then centrifuged at 3000 r/min for 10 minutes (centrifugation radius: 10 mm). Serum was separated, followed by enzyme-linked immunosorbent assay (ELISA) to determine interleukin (IL)-6 (ab178013, Abcam, Cambridge, United Kingdom), tumor necrosis factor alpha (TNF-α) (ab181421, Abcam), and IL-1β (ab214025, Abcam) levels, as well as serum S100β (ab234573, Abcam) and neuron-specific enolase (NSE) (ab217778, Abcam) concentrations.

### Electrochemiluminescence

The 5 mL of fasting venous blood collected preoperatively was subjected to plasma and serum separation within one hour. Serum myocardial enzyme indicators such as creatine kinase-muscle and brain isoenzyme (CK-MB), myoglobin (Mb), N-terminal B-type natriuretic peptide (NT-proBNP), and high-sensitivity cardiac troponin T (Hs-cTnT) were assessed by a Siemens ADVIA CENTAUR XP automatic chemiluminescence immunoassay analyzer (Siemens Diagnostics, Tarrytown, New York, United States of America). All kits utilized were from Siemens (Erlangen, Germany).

### Echocardiography

Echocardiographic examinations were performed using Philips IE 33 echocardiographic system to assess left ventricular end-diastolic dimension (LVEDD), LVEF, and left atrial diameter (LAD).

### Blood Coagulation Detection

A fully automatic blood coagulation analyzer (SF-8200C, Succeeder, Beijing, China) was utilized to determine the coagulation indicators preoperatively, and at T_6_, T_7_, and 24 hours postoperatively, including fibrinogen (FIB), thrombin time (TT), activated partial thromboplastin time (APTT), and prothrombin time (PT).

### Cognitive Function Assessment

As previously described^[33,34]^, the patients’ cognitive function was assessed using the Mini-Mental State Examination (MMSE) and Montreal Cognitive Assessment (MoCA) scores before and seven days after surgery, respectively. According to the MMSE scores, 27 to 30 points were seen as normal, 21 to 26 points as mild cognitive impairment, 10 to 20 points as moderate cognitive impairment, and zero to nine points as severe cognitive impairment. The MoCA score had a total score of 30 points, with 26 points and above indicating normal, and a higher score representing a better cognitive function.

### Statistical Analysis

Sample size estimation was performed using the G*Power 3.1.9.7 software (University of Düsseldorf), and the included sample size met the requirements of the independent sample *t*-test, Mann-Whitney U test, and Chi-square test. Data were analyzed and graphed using IBM Corp. Released 2012, IBM SPSS Statistics for Windows, version 21.0, Armonk, NY: IBM Corp. and GraphPad Prism 6.0 (GraphPad Software, San Diego, California, United States of America). The Kolmogorov-Smirnov test was used to test for normal distribution, and measurement data that conformed to normal distribution were expressed as mean ± standard deviation. Comparisons between two groups were conducted using the independent sample *t*-test, and comparisons between time points within groups were made using repeated measures analysis of variance. Measurement data that were not normally distributed were presented as median (minimum, maximum), and inter-group comparisons were made using the Mann-Whitney U test. Counting data were expressed as cases and percentages, and the Chi-square test was adopted for comparisons between groups. *P* was a two-sided test. The level of significance was *P* < 0.05.

## RESULTS

### Clinical Baseline Characteristics of the Two Groups

There were no statistical differences between the two groups in terms of clinical baseline data such as age, sex, CPB time, aortic occlusion time, heart functional classification, preoperative CK-MB, Mb, NT-proBNP, Hs-cTnT, LVEF, LVEDD, LAD, education background, comorbidities, operation approach, operation time, bleeding volume, intraoperative red blood cell usage, and plasma usage (all *P* > 0.05) (Table 1).

**Table 1:** Comparisons of clinical baseline characteristics.

Items		Control group (n = 65)	Study group (n = 65)	*P*-value
Sex (male/female)		42/23	39/26	0.587
Age (years)		48.59 ± 6.14	50.27 ± 7.49	0.164
BMI (kg/m^2^)		21.85 ± 2.75	22.16 ± 3.17	0.553
Education length (year)		8.52 ± 2.13	8.94 ± 2.39	0.292
Preoperative heart functional classification (case)	Class II	17 (26.15)	20 (30.77)	0.560
	Class III	48 (73.85)	45 (69.23)	
Preoperative CK-MB (ng/mL)		2.23 ± 1.21	2.32 ± 1.27	0.680
Preoperative Mb (ng/mL)		39.85 ± 11.54	42.15 ± 12.59	0.280
Preoperative NT-proBNP (pg/mL)		2361.24 ± 594.48	2514.12 ± 623.91	0.155
Preoperative Hs-cTnT (pg/mL)		113.26 ± 29.85	121.07 ± 30.22	0.141
Extracorporeal circulation time (min)		109.12 ± 12.59	113.57 ± 15.33	0.073
Aortic occlusion time (min)		75.19 ± 17.49	71.24 ± 16.85	0.192
Operation time (min)		285.63 ± 20.92	291.48 ± 21.42	0.118
Bleeding volume (mL)		515.28 ± 78.43	497.86 ± 85.44	0.228
Intraoperative red blood cell usage (U)		2.77 ± 0.81	2.65 ± 0.75	0.382
Intraoperative plasma usage (mL)		449.26 ± 95.36	419.85 ± 96.39	0.083
Operation approach (cases)	Mitral valve replacement	48 (73.85)	53 (81.54)	0.574
	Aortic valve replacement	7 (10.77)	5 (7.69)	
	Aortic and mitral valve replacement	10 (15.38)	7 (10.77)	
Comorbidities (cases)	Hypertension	12 (18.46)	16 (24.62)	0.775
	Diabetes	5 (7.69)	7 (10.77)	
	Cerebrovascular diseases	6 (9.23)	5 (7.69)	
LVEF (%)		58.63 ± 6.35	59.02 ± 8.15	0.761
LVEDD (mm)		53.12 ± 8.96	54.12 ± 8.46	0.514
LAD (mm)		47.25 ± 4.25	47.63 ± 5.17	0.648

BMI=body mass index; CK-MB=creatine kinase-muscle and brain isoenzyme; Hs-cTnT=High-sensitivity cardiac troponin T; LAD=left atrial diameter; LVEDD=left ventricular end-diastolic dimension; LVEF=left ventricular ejection fraction; Mb=myoglobin; NT-proBNP=N-terminal B-type natriuretic peptide

Counting data were depicted as cases and percentages. The Chi-square test was applied for intergroup comparisons. Measurement data conforming to normal distribution were expressed as mean ± standard deviation, and the independent sample *t*-test was adopted for comparisons between two groups

### Comparison of Index of Intraoperative Coagulation Function of Patients in Both Groups

**Fig. 1:**
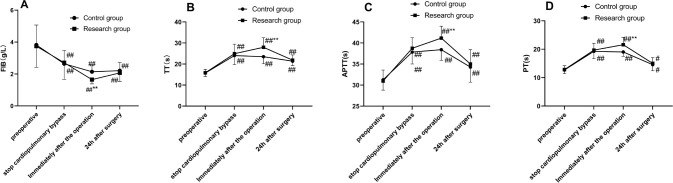
Comparisons of coagulation indicators between the two groups at each time point. A - D) Fibrinogen (FIB), thrombin time (TT), activated partial thromboplastin time (APTT), and prothrombin time (PT) levels were measured using an automatic coagulation analyzer. Data were expressed as mean ± standard deviation. Comparisons between the two groups were performed using independent sample t-test, and comparisons between time points within the two groups were conducted using repeated measures analysis of variance. Compared with the control group: ** P < 0.01. Compared with the preoperative period: ^#^P < 0.05 and ^##^P < 0.01.

During the preoperative period, at the cessation of CPB and 24 hours postoperatively, no prominent differences were observed in FIB, TT, APTT, and PT levels between the two groups (all *P* > 0.05). Compared with the preoperative period, TT, APTT, and PT levels were elevated, and FIB level was decreased at T_7_ and 24 hours postoperatively in both groups, and the changes were more notable in the research group at T_7_ (all *P* < 0.05, Figure 1A-D). These results suggested that patients in the research group with O3-AHT had more severe intraoperative coagulation dysfunction compared with those with banked blood transfusion in the control group.

### Comparison of Postoperative Inflammatory Response of Patients in Both Groups

**Fig. 2:**
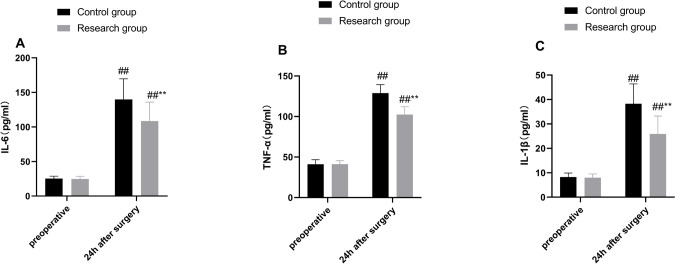
Comparisons of preoperative and postoperative 24-hour serum inflammatory factor levels between the two groups. Serum (A) interleukin (IL)-6, (B) tumor necrosis factor alpha (TNF-α), and (C) IL-1β levels were determined by enzyme-linked immunosorbent assay. Data were depicted as mean ± standard deviation. Independent sample t-test was utilized for comparisons between two groups, and paired t-test was employed for comparisons within the groups. Compared with the control group: ** P < 0.01. Compared with preoperative period: ^##^P < 0.01.

ELISA results demonstrated that the differences in preoperative IL-6, TNF-α, and IL-1β levels between the two groups were not statistically significant (all *P* > 0.05). In addition, serum IL-6, TNF-α, and IL-1β levels were remarkably elevated 24 hours postoperatively compared to their preoperative levels in both groups (all *P* < 0.01, Figure 2A-C), and the levels were notably diminished in the research group *vs.* the control group (all *P* < 0.01, Figure 2A-C). The results indicated that both groups had mild inflammatory response 24 hours after operation, but in comparison to patients with banked blood transfusion, patients who received O3-AHT had decreased serum IL-6, TNF-α, and IL-1β levels 24 hours after operation.

### Comparison of Oxygen Metabolism in Brain Tissues of Patients in Both Groups

**Fig. 3:**
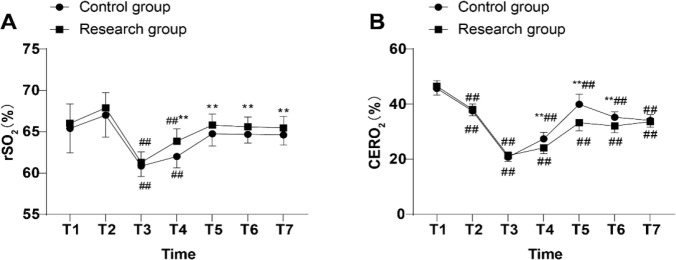
Comparisons of regional cerebral oxygen saturation (rSO_2_) and cerebral oxygen extraction rate (CERO_2_) at each time point between the two groups. Pre-anesthesia induction (T_1_), 10 minutes after anesthesia induction (T_2_), after ascending aorta occlusion (T_3_), 10 minutes after cooling to 30°C (T_4_), immediately after rewarming to 37°C (T_5_), at the end of CPB (T_6_), and immediately after operation (T_7_). An rSO_2_ monitor was used to measure rSO_2_ value, followed by blood gas analysis and calculation of CERO_2_. The data were expressed as mean ± standard deviation, independent sample t-test was utilized for comparisons between the two groups, and repeated measures analysis of variance for comparisons between each time point within the two groups. Compared with the control group: **P < 0.01. Compared with T1: ^##^P < 0.01.

The rSO_2_ values at T_3_ and T_4_ were significantly decreased in both groups compared to T_1_ in their respective groups (all *P* < 0.01, Figure 3A). The differences in rSO_2_ values at the T_1_, T_2_, and T_3_ were not statistically significant in both groups (all *P* > 0.05), whereas the values at time points T_4_, T_5_, T_6_, and T_7_ were raised in the patients of the research group relative to the control group (all *P* < 0.01, Figure 3A). CERO_2_ values of the patients in both groups at all-time points were dramatically reduced compared to T_1_ within their respective groups (all *P* < 0.01, Figure 3B); the differences in CERO_2_ values of the patients between the two groups at the T_1_, T_2_, T_3_, and T_7_ were not statistically significant (all *P* > 0.05); and the CERO_2_ values of the patients in the research group at T_4_, T_5_, and T_6_ were lower than those of the control group (all *P* < 0.01, Figure 3B). The aforementioned results manifested that relative to the banked blood transfusion, patients who received O3-AHT had improved intraoperative cerebral oxygen metabolism, which is protective against the occurrence of cerebral oxygen supply-demand imbalance.

### Comparison of Postoperative Cognitive Dysfunction in Patients in Both Groups

**Fig. 4:**
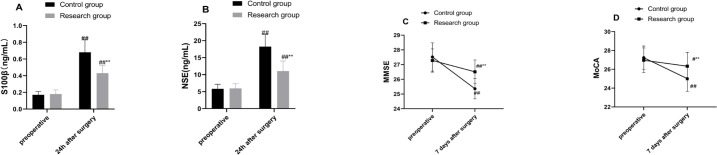
Comparisons of serum S100β and neuron-specific enolase (NSE) concentrations and Mini-Mental State Examination (MMSE) and Montreal Cognitive Assessment (MoCA) scores between the two groups. Enzyme-linked immunosorbent assay to measure serum S100β and NSE concentrations, as well as MMSE and MoCA scores, to assess the cognitive functions of the patients. Data were depicted as mean ± standard deviation, independent sample t-test was conducted for comparisons between two groups, and paired t-test for comparisons within groups. Compared with the control group: **P < 0.01. Compared with preoperative period: ^#^P < 0.05 and ^##^P < 0.01.

As reflected by ELISA results, the differences in S100β and NSE levels between the two groups did not demonstrate statistical significance preoperatively (all *P* > 0.05), whereas the levels in both groups were distinctly higher 24 hours postoperatively compared to preoperatively (all *P* < 0.01, Figure 4A-B). In addition, serum S100β and NSE levels in the research group were substantially reduced compared to the control group (all *P* < 0.01, Figure 4A-B). These results indicated that compared with banked blood transfusion, O3-AHT reduced the levels of brain injury markers in patients at 24 hours postoperatively. Further analyses of the MMSE and MoCA scores in the two groups unveiled that the differences in preoperative MMSE and MoCA scores in the two groups were not statistically significant (all *P* > 0.05), but the scores in both groups were lower than the preoperative ones at seven days postoperatively (all *P* < 0.05, Figure 4C-D), and were both hoisted in the research group relative to the control group (all *P* < 0.01, Figure 4C-D). Overall, both groups had decreased cognitive function 24 hours after operation, but patients with O3-AHT had attenuated postoperative cognitive dysfunction compared with patients who received banked blood transfusion.

### Comparison of Transfusion Complications in Patients in Both Groups

Complications of transfusion occurred in both groups and were recorded and analyzed. The overall complication incidence in control group was 4.62%, slightly higher than the research group (3.08%), but failed to achieve any statistically significant difference (*P* > 0.05, Table 2).

**Table 2:** Comparison of transfusion complications in patients in both groups.

Group	Allergic reaction	Hemolytic reaction	Non-hemolytic febrile transfusion reaction	Reaction after massive blood transfusion	Sum
Control group (N = 65)	1 (1.54)	0 (0.00)	1 (1.54)	1 (1.54)	3 (4.62)
Study group (N = 65)	0 (0.00)	1 (1.54)	1 (1.54)	0 (0.00)	2 (3.08)
*P*-value					0.648

## DISCUSSION

CPB represents the established method for surgical aortic valve replacement, recognized as the gold standard, and is deemed safe and linked to a low mortality rate^[35]^. Nevertheless, in its extreme manifestation, CPB stimulates coagulation abnormalities and systemic inflammatory responses that can result in adverse clinical consequences^[5]^. A recent review reported an increased association of CPB with unexpected consequences for pharmacokinetic parameters in children undergoing surgery for congenital heart disease in the past 10 years^[36]^. Notably, O3-AHT has been documented to enhance blood circulation and tissue oxygenation to essential organs^[37]^. Our study revealed that the application of O3-AHT in patients undergoing valve replacement with CPB might be beneficial, as evidenced by the improved brain tissue oxygen metabolism during the operation, decreased 24-hour postoperative inflammatory responses, and improved cognitive dysfunction.

Medical ozone can reduce lung inflammation, inhibit viral replication, prevent microvascular thrombosis, and modulate lung circulation and oxygenation^[38]^. It has been documented that TT, APTT, and PT levels are elevated in patients undergoing ozone therapy, and O3-AHT has been shown to effectively mitigate chemotherapeutic enteritis and reduce blood hypercoagulability in patients^[39]^. Ozone therapy stabilizes hepatic metabolism and normalizes the affinity and FIB levels and prothrombin in infected patients^[40]^. Similarly, our study unveiled that TT, APTT, and PT were all increased, and FIB was decreased at the immediate postoperative and 24-hour postoperative periods, with the changes more pronounced in patients receiving O3-AHT at the immediate postoperative period. The result on coagulation function also shows that patients in the research group receiving O3-AHT had more severe coagulation dysfunction compared to the control group in the immediate postoperative period, which may be explained by the fact that CPB has a certain effect on the coagulation function of patients. The blood transfused to patients was salvaged using O3-AHT and underwent a series of steps, including centrifugation, filter, washing, and concentration before transfusion, which affect the coagulation function of patients in a transfusion blood volume dependent manner^[41]^. But in this study, no perioperative coagulation disorder caused by coagulation reduction was observed. Systemic ozone therapy appears to be beneficial in regulating inflammation, enhancing immunity, and offering protections against acute coronary syndromes and ischemia-reperfusion injury^[42]^. O3-AHT regulates inflammation by influencing crucial cytokine expression levels associated with gout, such as serum IL-8 level^[17]^. The utilization of O3-AHT in the comprehensive management of acute soft-tissue infections in diabetic patients enables substantial reductions in wound inflammation and regeneration phase duration by suppressing IL-8 and IL-10 overproductions and promoting basic fibroblast growth factor and its receptor expression^[43]^. Intriguingly, our study manifested that both groups had mild inflammatory response 24 hours after operation, but the 24-hour postoperative serum levels of pro-inflammatory cytokines IL-6, TNF-α, and IL-1β were decreased in patients receiving O3-AHT when compared with patients receiving banked blood transfusion. The probable reason is that ozone targets inflammatory mechanisms by acting on more intricate intracellular pathways to modulate the interaction between nuclear factor erythroid-2-related factor 2/nuclear factor-κB (or Nrf2/NF-κB) and the mitochondria-associated inflammasome NOD-like receptor thermal protein domain associated protein 3 (or NLRP3), which in turn leads to reductions in serum inflammatory markers, whose role in viral diseases and other inflammatory conditions has been extensively documented^[44,45]^.

The assessment of oxygen consumption and supply in brain tissues serves as a crucial measure of normal aerobic metabolism in the brain, and monitoring the equilibrium between the supply and demand of cerebral oxygen during surgical anesthesia is essential for safeguarding brain function^[22]^. SjvO_2_ and CERO_2_ are pivotal indicators for the evaluation of cerebral oxygen metabolism^[46,47]^. Exposure to a combination of oxygen and ozone markedly enhances both mitochondrial activity and oxygen consumption rate^[48]^. Innovatively, our study found that compared to banked blood transfusion, patients receiving O3-AHT had improved intraoperative cerebral oxygen metabolism, favorable to the prevention of cerebral oxygen supply-demand imbalance. Ozone therapy has demonstrated significant efficacy in both ischemic and hypometabolic brain syndromes, such as stroke or radiation-induced brain injury, and appears to be effective in the restoration of damaged brain tissues^[49,50]^. Furthermore, medical ozone autologous blood transfusion therapy can activate erythrocytes, increase adenosine triphosphate and 2.3-diphosphoglyceric acid levels in erythrocytes, facilitate erythrocyte metabolism, and improve the oxygen-carrying and capillary-passing capacities of erythrocytes. Additionally, it also increases erythrocyte rheology and promotes the release of oxygen from erythrocytes, thus increasing the supply of oxygen to the body’s tissues^[51]^.

At three and seven days after treatment, there are prominent reductions in serum NSE and S100β levels of patients who undergoing O3-AHT plus Xingnaojing injection^[52]^. Consistent with prior research, our study findings indicated that patients receiving O3-AHT exhibited decreased S100β and NSE levels compared to those receiving banked blood transfusion. Oxygen-ozone therapy regulates various physiological processes such as immune, inflammatory response, metabolism, oxidation, microbiota, and regenerative mechanisms compromised in cognitive frailty^[53]^. A study by Abeer E El-Mehi demonstrated the advantageous impact of ozone in ameliorating the neurodegenerative changes in the cerebral cortex of elderly rats^[54]^. Ozone has been shown to inhibit amyloid-β precursor protein (APP)/amyloid-β peptides (or Aβ) production and enhance cognitive function in an APP/presenilin 1 (or PS1) transgenic mouse model^[55]^. Interestingly, our study revealed that MMSE and MoCA scores of patients receiving O3-AHT were higher than those of the patients accepting banked blood transfusion, suggesting that patients receiving O3-AHT or banked blood transfusions both had reduced postoperative cognitive dysfunction, but patients in the research group receiving O3-AHT had attenuated postoperative cognitive dysfunction compared to banked blood transfusions. In addition to that, we also recorded the transfusion complications of both groups, and the results showed that the complication incidence of the control group and research group was 4.62% and 3.08%, respectively. But the complication incidence between the two groups showed no significant difference, suggesting that the O3-AHT technique does not increase the risk of transfusion complications in patients undergoing valve replacement with CPB, as compared to banked blood transfusion.

### Limitations

Nevertheless, there are several limitations that need to be considered. Firstly, the sample size of this study was limited, and it inadequately represented the patient population. Secondly, despite the uniform postoperative management on all patients by a consistent team of surgeons, the surgeons were not informed of the intraoperative changes in rSO_2_, and no monitoring of rSO_2_ was conducted postoperatively, leading to the facts that cerebral deoxygenation could potentially transpire during this phase, impacting the postoperative cognitive function of patients probably. Thirdly, the inflammatory response is a multifaceted process, in which calcitonin and complement factors, including C3a and C5a, play crucial roles during inflammatory responses in CPB. However, these factors related to inflammation were not detected under the prevailing conditions. Fourthly, transfusion of banked blood has certain effects on inflammatory response and postoperative adverse effects in patients undergoing valve replacement with CPB when compared with autologous blood transfusion. Additionally, there exists heterogeneity in patients undergoing valve replacement with CPB. This study did not stratify individual patient differences and failed to compare the effectiveness of the O3-AHT across different populations. Furthermore, potential confounding factors, such as preoperative complications, postoperative treatment modalities, and perioperative nursing interventions, may affect the study’s results. Future study includes a third group including the patients transfused with autologous blood prepared preoperatively without O3-AHT or having the strategy of autologous blood transfusion without O3-AHT for the control group would be interesting, and this will certainly be a future direction for us. In subsequent studies, we plan to enlarge the sample size and prolong postoperative rSO_2_ monitoring to enhance the credibility of the findings.

## CONCLUSION

All in all, our study highlighted that the application of O3-AHT on patients undergoing valve replacement with CPB might be beneficial, as manifested by enhanced intraoperative brain tissue oxygen metabolism, reduced 24 hours postoperatively inflammatory response, and reduced cognitive dysfunction.
